# Fecal Immunochemical Test Screening Status, Stage Distribution, and Overall Survival in Colorectal Cancer: A Propensity Score-Matched Cohort Study from a Regional Hospital in Taiwan

**DOI:** 10.3390/diagnostics16142151

**Published:** 2026-07-09

**Authors:** Mei-Wen Chen, Po-Ju Lin, Jing-Jim Ou, Cheng-Shyong Chang

**Affiliations:** 1 Cancer Administration and Coordination Center, Chang Bing Show Chwan Memorial Hospital, Changhua 505, Taiwan; 2 Department of Information Management, National Open University, New Taipei 247, Taiwan; 3 Department of Radiation Oncology, Chang Bing Show Chwan Memorial Hospital, Changhua 505, Taiwan; 4 Department of Colon and Rectal Surgery, Chang Bing Show Chwan Memorial Hospital, Changhua 505, Taiwan; jingjimou@gmail.com; 5 Department of Hematology and Oncology, Chang Bing Show Chwan Memorial Hospital, Changhua 505, Taiwan; cs4816@gmail.com

**Keywords:** fecal immunochemical test, colorectal cancer screening, clinicopathological features, overall survival, propensity score matching

## Abstract

**Background/Objectives**: Fecal immunochemical testing (FIT)-based screening for colorectal cancer (CRC) has been associated with reductions in CRC mortality and improvements in early-stage disease detection; however, most related evidence has been derived from large national registries. The present study investigated associations of FIT screening status with overall survival (OS) and several clinicopathological characteristics in a regional hospital setting. **Methods**: After propensity score matching, 912 patients with pathologically confirmed colorectal adenocarcinoma diagnosed between 2010 and 2025 at a regional hospital in Taiwan were included. Patients were categorized into screened (*n* = 304) and nonscreened (*n* = 608) groups by using propensity score matching based on age as a continuous variable, sex, and stage. OS and clinicopathological characteristics in each group were compared. Multivariable Cox regression was performed using a primary reduced model, an exploratory full model, and multiple imputation analysis. **Results**: Screened patients demonstrated better 15-year OS (58.38% vs. 45.80%; log-rank *p* = 0.005), especially those with early-stage disease (*p* = 0.001). FIT screening was independently associated with improved OS in the primary reduced model (hazard ratio [HR] = 0.63; 95% confidence interval [CI]: 0.45–0.88; *p* = 0.006) and multiple imputation analysis (HR = 0.68; 95% CI: 0.50–0.91; *p* = 0.010). Independent adverse prognostic factors were male sex, late-stage disease, perineural invasion, tumor obstruction, and abnormal pretreatment carcinoembryonic antigen level. **Conclusions**: FIT screening was associated with better long-term OS in a stage-balanced cohort, but interpretation is limited by the use of OS rather than CRC-specific survival and residual age imbalance. Improving FIT participation, timely colonoscopy follow-up, and complete pathological staging remains essential for CRC outcomes.

## 1. Introduction

Colorectal cancer (CRC) is one of the most common and deadly malignancies worldwide, ranking third in incidence and second in cancer-related mortality, with approximately 1.93 million new cases and 904,000 deaths reported in 2022 [1,2]. In Taiwan, CRC has consistently ranked as either the first or the second most common cancer. According to the 2023 national cancer registry report, 19,074 individuals received diagnoses of new-onset CRC in 2023 [3], which made CRC the second most common cancer in that year. Additionally, CRC was the third leading cause of cancer-related death in Taiwan in 2022 [3]. Despite major advances in surgery and systemic therapy, survival remains substantially poorer for patients with late-stage disease than for those with early-stage disease [2]. Because stage at diagnosis is one of the strongest determinants of long-term survival, population-based screening has become a central strategy in CRC control worldwide [4].

Fecal immunochemical testing (FIT), a noninvasive antibody-based form of fecal occult blood testing, has been widely adopted as a cost-effective screening tool for CRC in population-based programs across Asia, Europe, and North America. As a quantitative immunochemical assay, FIT has largely replaced guaiac-based fecal occult blood testing because of its superior sensitivity and specificity for detecting colorectal neoplasia [5]. Systematic reviews and large cohort studies have demonstrated that FIT-based screening is associated with reductions in CRC-related mortality and that FIT-based screening better facilitates earlier-stage diagnosis and curative-intent treatment, although the magnitude of benefit varies across study designs and health-care settings [6,7]. In Taiwan, biennial stool-based screening has been available for quite some time. The National Colorectal Cancer Screening Program began offering screening services to individuals aged 50–69 years in 2004; in 2013, the service was extended to individuals aged 70–74 years. National registry analyses have reported improved stage distribution among screening-detected CRC cases and a measurable decline in CRC mortality since the screening services were first provided [8]. A multidatabase cohort study involving more than 1.5 million Taiwanese individuals demonstrated that FIT-based screening was associated with a 33% reduction in CRC incidence and a 47% reduction in CRC-specific mortality, with a clear dose–response relationship between cumulative screening exposure and survival benefit [9]. National program data additionally indicate that regular screening, together with timely treatment, reduced the incidence of late-stage CRC by 29% and the rate of overall CRC mortality by 35%, with 5-year survival exceeding 90% among patients who received a diagnosis at stage 0 or I [10]. Reflecting Taiwan’s continued commitment to early detection, the Health Promotion Administration expanded free biennial FIT screening in 2025 to include individuals aged 45–74 years, with individuals aged 40–44 years with a family history of CRC also being eligible for free screening [10].

Although the survival benefit of population-based FIT screening has been well established at the national level, relatively little attention has been directed toward its association with real-world clinicopathological and survival outcomes after adjustment for baseline clinical differences in regional hospital settings. In particular, institution-level evidence of the associations of FIT screening with histological grade, lymphovascular invasion (LVI), perineural invasion (PNI), pretreatment carcinoembryonic antigen (CEA) level, tumor obstruction or perforation, and various molecular alterations remains limited. These pathological and molecular features are clinically important not only because they predict prognosis but also because they influence treatment planning, recurrence risk assessment, and precision oncology strategy selection. Furthermore, although national screening programs often demonstrate a clear stage shift, stage distribution after stage-inclusive propensity score matching (PSM) in a single regional hospital should be interpreted cautiously.

Most available evidence has been derived from large national registries or academic medical centers [11,12,13,14,15,16], leaving a notable literature gap regarding how FIT screening translates into real-world pathological and survival outcomes at the regional hospital level. Patient populations in regional settings may differ substantially from those represented in national registries or academic medical centers in terms of socioeconomic background, comorbidity burden, health literacy, screening adherence, referral patterns, and health-care-seeking behavior [17,18]. This gap is particularly relevant in Taiwan, where regional hospitals serve heterogeneous rural and urban populations and play a central role in cancer diagnosis, treatment, and follow-up. Therefore, studies analyzing data from regional hospitals are essential for determining whether FIT screening status is independently associated with clinicopathological outcomes and survival. Such studies should use appropriate matching and multivariable adjustment to ensure fair comparisons, with the caveat that stage-inclusive matching may attenuate the population-level stage-shift benefit typically associated with FIT screening.

To address this gap, we conducted a retrospective cohort study at a regional hospital in Taiwan. We compared clinicopathological and molecular characteristics in screened and nonscreened patients with pathologically confirmed colorectal adenocarcinoma between 2010 and 2025. To reduce confounding in the survival analysis, we applied 1:2 PSM to balance screened and nonscreened patients on the basis of age, sex, and stage. Because tumor stage may represent an intermediate factor on the causal pathway between FIT screening and survival, this stage-inclusive matched analysis was not designed to estimate the total survival benefit of FIT screening. Instead, it was intended to determine whether FIT screening status was associated with long-term OS among patients with comparable stage distribution. The primary outcomes of interest included histological grade, LVI, PNI, pretreatment CEA level, tumor obstruction, tumor perforation, mutation (BRAF, KRAS, and NRAS) status, and long-term overall survival (OS). The distribution of early-stage versus late-stage disease was confirmed to be balanced in the matched groups. Kaplan–Meier and Cox proportional hazards regression analyses were performed to determine whether FIT screening was associated with improved clinicopathological characteristics and long-term survival within this stage-balanced cohort.

## 2. Methods

### 2.1. Study Design and Data Extraction

This retrospective cohort study was conducted at Chang Bing Show Chwan Memorial Hospital, a regional hospital in Changhua County, Taiwan. The study protocol was approved by the institutional review board of the study hospital (protocol number: 1150111). The requirement for informed consent was waived because of the retrospective design of this study and the use of deidentified registry data. Patient data were integrated from three complementary institutional databases: (1) the CRC screening registry, which documents each patient’s FIT participation history and test results obtained prior to CRC diagnosis, reflecting true population-based screening rather than post-diagnosis surveillance; (2) the hospital cancer registry, which contains demographic, clinical, pathological, and staging information; and (3) the oncology case management database, which offers longitudinal follow-up data on treatment and survival status. Datasets were linked using each patient’s unique medical record number.

**Figure 1:**
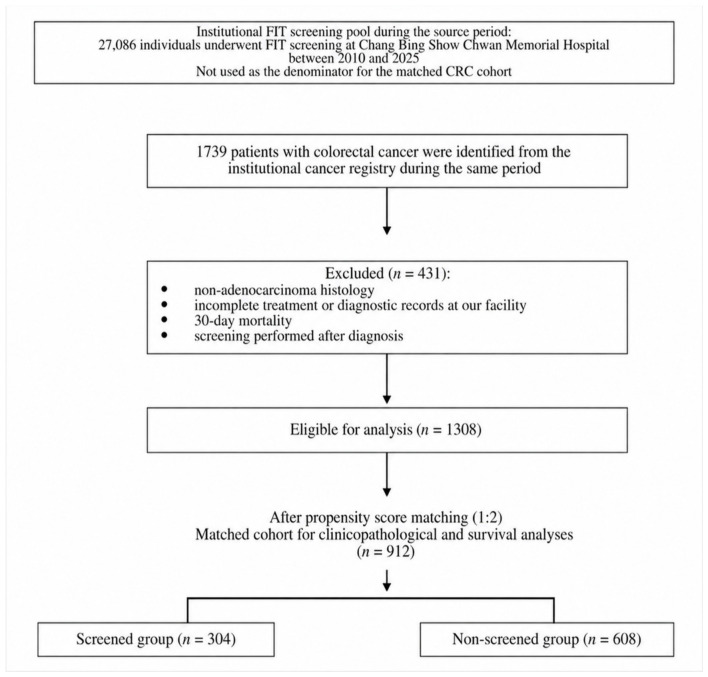
Flowchart depicting patient selection. Potentially eligible patients were those with colorectal cancer who underwent FIT screening at Chang Bing Show Chwan Memorial Hospital between 2010 and 2025. The 27,086 individuals represent the institutional FIT screening pool during the study period and were not used as the denominator for the matched CRC cohort.

This study included patients with pathologically confirmed colorectal adenocarcinoma diagnosed between January 2010 and December 2025. Patients with unavailable FIT screening-status information or incomplete key clinicopathological data were excluded. Of 1739 potentially eligible patients, 431 were excluded for the following reasons: histological ruling out of adenocarcinoma, incomplete treatment or diagnostic records, death within 30 days of diagnosis, or FIT after cancer diagnosis. Thus, the study cohort comprised 1308 patients (Figure 1).

FIT screening status was defined by the presence of at least one documented institutional record of FIT screening before CRC diagnosis. FIT screenings conducted outside of the study hospital may not have been fully captured; this represents a limitation of the present study and is further discussed in the Limitations section. After PSM, the final analytic cohort comprised 304 screened patients and 608 nonscreened patients (*n* = 912).

Clinical variables were age at diagnosis, sex, lifestyle factors (alcohol, cigarette, and betel nut consumption). Disease-specific variables were primary tumor site, histological grade, stage (per American Joint Committee on Cancer criteria), pretreatment CEA level, LVI, PNI, tumor obstruction and perforation, mutation (*BRAF*, *KRAS*, and *NRAS*) status, comorbidities, number of primary cancers, and survival status. Stage was defined according to the available AJCC staging information in the institutional cancer registry, incorporating clinical and pathological staging data when available. Molecular testing was performed selectively in patients with metastatic or advanced disease and is therefore subject to stage-dependent ascertainment bias, as noted in the Limitations section. OS was defined as the interval between CRC diagnosis and death from any cause or the last follow-up (12 February 2026). CRC-specific death was not analyzed because reliable cause-of-death information was not consistently available in the institutional registry database. Therefore, OS was selected as the primary survival endpoint in this study.

### 2.2. Statistical Analysis

Clinicopathological characteristics were summarized using descriptive statistics. Continuous variables were compared using an independent-samples *t* test and are presented as mean ± standard deviation values. By contrast, categorical variables were compared using Pearson’s chi-square test or Fisher’s exact test and are presented as frequencies and percentages. All tests were two-sided, with *p* < 0.05 considered statistically significant.

Propensity scores were estimated using binary logistic regression with FIT screening status as the dependent variable and age as a continuous variable, sex, and stage as matching covariates. These covariates were selected because age, sex, and tumor stage are established prognostic factors for colorectal cancer survival and may also differ systematically between screened and nonscreened patients. We acknowledge that tumor stage may partly mediate the effect of FIT screening on survival. Therefore, stage was included in the propensity score model not to estimate the total effect of FIT screening through earlier-stage detection, but to create a conservative stage-balanced comparison and to evaluate whether FIT screening status retained prognostic relevance among patients with similar disease stage. Age was modeled as a continuous variable to improve matching precision, whereas age-group categories (<65, 65–75, and >75 years) were retained for descriptive comparison and subsequent age-stratified survival analysis. Patients were matched at a 1:2 ratio (screened/nonscreened) by using nearest-neighbor matching without replacement. Covariate balance in the propensity score-matched cohort was assessed using postmatching baseline characteristics and standardized mean differences (SMDs). An absolute SMD of less than 0.10 was considered indicative of adequate covariate balance, consistent with established methodological recommendations for propensity score analyses.

Postmatching covariate balance was re-examined to identify variables with residual imbalance. Although mean age and stage were well balanced after matching, residual imbalance remained in age group, sex, betel nut chewing, histological grade, pretreatment CEA level, and several comorbidity variables, most notably an SMD of 0.305 for age-group distribution. We considered re-performing propensity score matching using a stricter caliper width or exact matching on age category to further reduce this imbalance; however, because the >75-year age stratum already comprised a limited number of patients (particularly in the screened group, *n* = 24), imposing stricter age-category matching criteria would have substantially reduced the effective sample size and statistical precision within this already sparse subgroup, without a proportionate gain in overall covariate balance. We recognize that, in principle, minimizing residual confounding should be prioritized over preserving sample size; in this instance, however, we judged that stricter age-category matching would have concentrated further loss of precision within the subgroup already most vulnerable to instability, potentially exchanging one source of bias for another rather than achieving a net improvement in balance.

In parallel, we also considered Inverse Probability of Treatment Weighting (IPTW) as an alternative to matching, given its ability to retain the full analytic sample and to use continuous propensity scores rather than discrete nearest-neighbor pairing. However, IPTW estimates can become unstable when extreme weights arise from small strata, and the >75-year age group in this cohort, particularly the screened subgroup (*n* = 24), represented such a sparse stratum. Reliable assessment of weight stabilization and truncation would require a full unmatched-cohort sensitivity analysis, which was beyond the prespecified matched-cohort analytic framework of the present study; we therefore retained 1:2 nearest-neighbor PSM as the primary analytic approach. Residual age-related confounding was instead addressed through two complementary strategies: entering age as a continuous covariate in the multivariable Cox regression models, for which postmatching balance was excellent (SMD = 0.009), and performing prespecified age-stratified survival analyses as descriptive robustness assessments. These analyses were used to examine whether the observed OS pattern was driven mainly by residual age-group imbalance, rather than as substitutes for exact age-category matching or IPTW. They suggested that the observed OS pattern was not explained solely by the excess proportion of patients aged >75 years in the nonscreened group; however, the findings should be interpreted cautiously because the screened subgroup aged >75 years was small.

Therefore, these variables were carefully considered in subsequent multivariable Cox regression models and sensitivity analyses to reduce the influence of residual confounding. The Results and Limitations sections further acknowledge that propensity score matching reduced, but did not completely eliminate, measured baseline imbalance.

To assess stage balance following PSM, tumor stage was dichotomized into early-stage (Stages 0–II) and late-stage (Stages III and IV) categories. Between-group comparability was evaluated using the chi-square test and is presented as odds ratios (ORs) with 95% confidence intervals (CIs).

OS was estimated using Kaplan–Meier analysis, with between-group differences evaluated using the log-rank test. Subgroup analyses by stage category and age group were performed.

To identify prognostic factors, univariate and multivariable Cox proportional hazards regression analyses were performed. Candidate variables included FIT screening status, age, sex, lifestyle factors, tumor stage, histological grade, LVI, PNI, obstruction, perforation, number of primary cancers, pretreatment CEA, and comorbidities (diabetes mellitus, hypertension, coronary artery disease, hepatitis B and C, end-stage renal disease, cerebrovascular accident, chronic obstructive pulmonary disease, chronic kidney disease, heart disease, and hyperlipidemia). Variables with *p* < 0.10 in univariate analysis or established clinical relevance were entered into the multivariable model. Notably, variables with fewer than 10 events or complete separation were excluded. Results are reported as hazard ratios (HRs) with 95% CIs; tied times were handled using the Breslow method. The proportional hazards assumption for the Cox regression models was assessed using Schoenfeld residuals with the cox.zph function in the R survival package. For the full multivariable Cox model, the global Schoenfeld residual test did not indicate a significant violation of the proportional hazards assumption (global *p* = 0.366). For the reduced Cox model, the global test was also nonsignificant (global *p* = 0.281), and no significant violation was observed for the main exposure variable, FIT screening status (*p* = 0.138). However, stage and tumor obstruction showed possible time-dependent effects in the reduced model. Because the global tests were nonsignificant and the main exposure variable satisfied the proportional hazards assumption, the Cox proportional hazards regression approach was considered acceptable for estimating the association between FIT screening status and overall survival.

Given high nonassessable rates for LVI (44–50%) and PNI (54–55%), two multivariable Cox models were prespecified. Missing data were not evenly distributed across variables. Missingness was mainly concentrated in pathological variables, particularly LVI and PNI, whereas most demographic, lifestyle, stage, comorbidity, and survival variables were complete or had relatively low missingness. Pretreatment CEA level and histological grade also contained some missing or nonassessable values and were therefore considered in the missing data strategy.

The primary reduced model was restricted to covariates with less than 20% missing data and excluded LVI and PNI. The second model was an exploratory full model that additionally incorporated these two variables. Tumor stage was used as a matching covariate in the propensity score model, and it is worth noting that stage may similarly lie along the causal pathway between FIT screening and survival, as discussed in the Introduction and Limitations sections. Stage was nonetheless retained for matching, rather than excluded on the same downstream grounds as LVI and PNI, for three reasons: it is routinely assessed at diagnosis, it was recorded in the great majority of the cohort, and it was needed to establish comparable baseline disease extent between the screened and nonscreened groups.

LVI and PNI, in contrast, are ascertained postoperatively from surgical pathology and had missing data in 44–55% of patients. This substantial missingness was the principal reason for excluding them from the primary reduced model. Their downstream biological timing relative to screening—discussed further in the Results and Discussion sections—is presented as an additional interpretive consideration, not as the main rationale for their exclusion.

A multiple imputation sensitivity analysis was conducted using the mice (Multivariate Imputation by Chained Equations) package in R, which handles missing data by generating multiple plausible imputed datasets through chained regression equations under the missing-at-random assumption (*m* = 20, predictive mean matching; pooled per Rubin’s rules), on the full matched cohort to assess robustness. The imputation model included FIT screening status, age, sex, lifestyle factors, tumor stage, histological grade, LVI, PNI, tumor obstruction, tumor perforation, number of primary cancers, pretreatment CEA level, comorbidities, survival time, and survival status. Imputed estimates from the Cox regression models were combined using Rubin’s rules, and the pooled hazard ratios, 95% confidence intervals, and *p* values were reported.

All analyses were performed in R version 4.5.1 (R Foundation for Statistical Computing, Vienna, Austria) by using the following open-source packages, all freely available through the Comprehensive R Archive Network (CRAN): MatchIt (version 4.7.2, for PSM), survival (version 3.8-6, for Kaplan–Meier and Cox analyses), survminer (version 0.5.1) and ggplot2 (version 4.0.0, for visualization), tableone (version 0.13.2, for covariate balance), and mice (version 3.18.0; Utrecht University, Utrecht, The Netherlands) for multiple imputation.

## 3. Results

### 3.1. Patient Selection and Cohort Characteristics

The patient selection process is depicted in Figure 1. Of the 1739 potentially eligible patients, 431 were excluded, leaving a final study cohort of 1308 patients with pathologically confirmed colorectal adenocarcinoma. Separately, 27,086 individuals underwent institutional FIT screening at Chang Bing Show Chwan Memorial Hospital during the study period; this figure represents the total institutional FIT screening pool and was not used as the denominator for the CRC cohort. After 1:2 PSM, the final matched cohort comprised 304 screened and 608 nonscreened patients (*n* = 912). Postmatching baseline characteristics are presented in Table 1. Because the available analytic dataset consisted of the propensity score-matched cohort, Table 1 presents the baseline clinicopathological characteristics of the matched screened and nonscreened groups and was used to evaluate postmatching comparability. The matching procedure achieved adequate balance for mean age and stage; however, residual imbalance remained for several variables, including age group, sex, betel nut chewing, and selected comorbidities. Therefore, postmatching comparability was interpreted cautiously, and clinically relevant variables were further considered in the multivariable Cox regression models to reduce the potential influence of residual confounding. For the early-stage versus late-stage analysis, 6 patients with unknown or unavailable stage data were further excluded, leaving 302 screened and 604 nonscreened patients.

Postmatching baseline clinicopathological characteristics are detailed in Table 1. Overall, PSM improved the comparability of several measured variables, including mean age, primary tumor site, stage, and most pathological characteristics. However, residual imbalance remained for age group, sex, betel nut chewing, pretreatment CEA level, histological grade, and selected comorbidities, as indicated by SMD values ≥ 0.10. Therefore, the postmatching baseline characteristics were interpreted as showing improved but incomplete covariate balance, and residual imbalance was further addressed through multivariable Cox regression models and sensitivity analyses. Mean age was nearly identical between the screened and nonscreened groups after matching (63.40 ± 8.80 vs. 63.50 ± 13.50 years; *p* = 0.880; SMD = 0.009). However, age-group distribution remained imbalanced (SMD = 0.305), reflecting a higher proportion of patients aged >75 years in the nonscreened group. Sex distribution also showed residual imbalance despite a borderline nonsignificant *p* value (male: 62.80% vs. 56.10%; *p* = 0.061; SMD = 0.138). Among lifestyle factors, alcohol use and cigarette smoking were acceptably balanced, whereas betel nut chewing showed residual imbalance (8.60% vs. 5.40%; *p* = 0.096; SMD = 0.123). Moreover, the screened and nonscreened groups did not differ significantly in terms of primary tumor site (distal: 74.30% vs. 73.40%; *p* = 0.811), mutation status (BRAF: *p* = 0.553; KRAS: *p* = 0.614; NRAS: *p* = 0.400), tumor obstruction (*p* = 0.759), tumor perforation (*p* = 0.148), LVI (*p* = 0.145), PNI (*p* = 0.447), or overall stage distribution (*p* = 0.413). Pretreatment CEA level showed a nonsignificant between-group difference but a residual SMD slightly above 0.10 (abnormal: 26.60% vs. 34.00%; *p* = 0.077; SMD = 0.114), and was therefore considered in the multivariable survival analysis.

Two clinicopathological variables differed significantly between the groups. Because tumor stage was itself a propensity-score matching variable, this comparison reflects a largely stage-independent evaluation of tumor biology. Histological grade was more favorable in the screened group, which had a higher proportion of well-differentiated tumors, than in the nonscreened group (30.30% vs. 19.60%; *p* = 0.001). Moderately differentiated tumors and tumors of unknown grade were more prevalent in the nonscreened group than in the screened group (moderately differentiated tumors: 61.00% vs. 54.90%; tumors of unknown grade: 14.60% vs. 8.20%). This stage-independent difference in histological grade suggests that screening-detected tumors may carry intrinsically more favorable biological features, rather than the difference simply reflecting an earlier stage at detection. By contrast, other tumor characteristics did not differ significantly between the screened and nonscreened groups, including LVI (*p* = 0.145), PNI (*p* = 0.447), tumor obstruction (*p* = 0.759), tumor perforation (*p* = 0.148), pretreatment CEA level (*p* = 0.077), BRAF mutation status (*p* = 0.553), KRAS mutation status (*p* = 0.614), NRAS mutation status (*p* = 0.400), and overall stage distribution (*p* = 0.413). In addition, the proportions of stage IV disease were similar between the screened and nonscreened groups (12.80% vs. 12.00%), suggesting no marked difference in the available stage-based indicator of metastatic disease burden after propensity score matching. Tumor size and detailed metastatic burden variables, such as number or sites of metastases, were not consistently available in the institutional registry and therefore could not be directly compared.

Age-group distribution also differed significantly between the two groups (*p* = 0.001), with the nonscreened group having a greater proportion of patients aged >75 years (23.00% vs. 7.90%) and the screened group having a greater proportion of patients aged 65–75 years (38.20% vs. 24.00%), consistent with the age eligibility range of Taiwan’s national FIT screening program. Although mean age was well balanced after PSM, the residual imbalance in age-group distribution indicates that age-related confounding could not be completely eliminated. Given the fixed cohort size and the already limited number of patients aged >75 years in the screened group (*n* = 24), re-matching with a stricter caliper width or exact age-category criteria was not pursued, as this would have further reduced statistical power in this vulnerable subgroup without a proportionate benefit. Therefore, age-stratified survival analyses were conducted as descriptive robustness assessments rather than as substitutes for exact age-category matching or IPTW. Age and sex were also retained in subsequent multivariable Cox regression models to further account for these residual differences. These analyses suggested that the overall OS pattern was not explained solely by the residual imbalance in patients aged >75 years; nevertheless, the findings should be interpreted cautiously because the number of screened patients aged >75 years was small.

### 3.2. Stage Distribution

**Figure 2:**
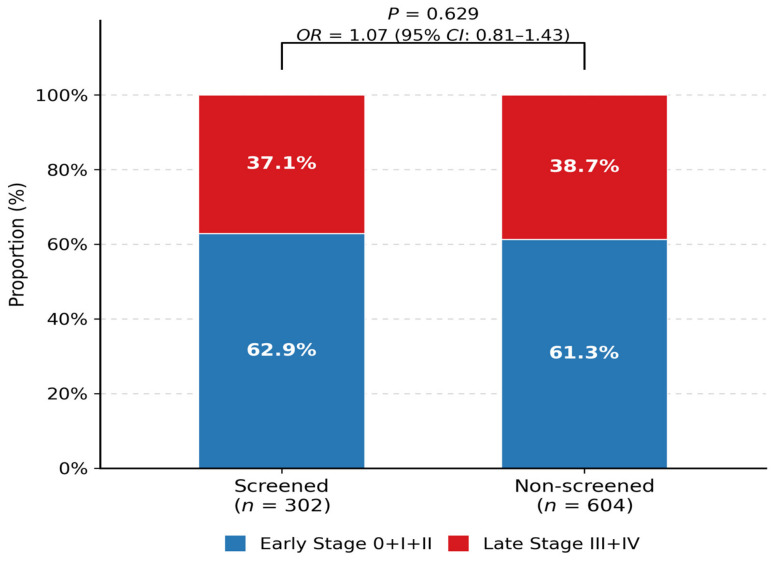
Stage distributions in the matched screened and nonscreened groups.

The distribution of early-stage versus late-stage disease in each group is presented in Table 2 and Figure 2. Among the 906 patients with known stage data, early-stage disease (Stages 0–II) was identified in 190 screened patients (62.90%) and 370 nonscreened patients (61.30%), whereas late-stage disease (Stages III and IV) was identified in 112 screened patients (37.10%) and in 234 nonscreened patients (38.70%). After stage-inclusive PSM, the early-stage versus late-stage distribution was comparable between the two groups, as expected by design (*p* = 0.629; OR for late-stage disease in nonscreened versus screened patients = 1.07; 95% CI: 0.81–1.43). Because stage was incorporated as a matching variable in the propensity score model, this postmatching comparison should not be interpreted as evidence for or against a population-level stage-shift effect of FIT screening. Rather, the result suggests that the matched cohort was balanced with respect to stage category. Accordingly, subsequent survival analyses should be interpreted as stage-balanced comparisons of screened and nonscreened patients, rather than as analyses of the total screening effect mediated through earlier-stage diagnosis.

### 3.3. OS

**Figure 3:**
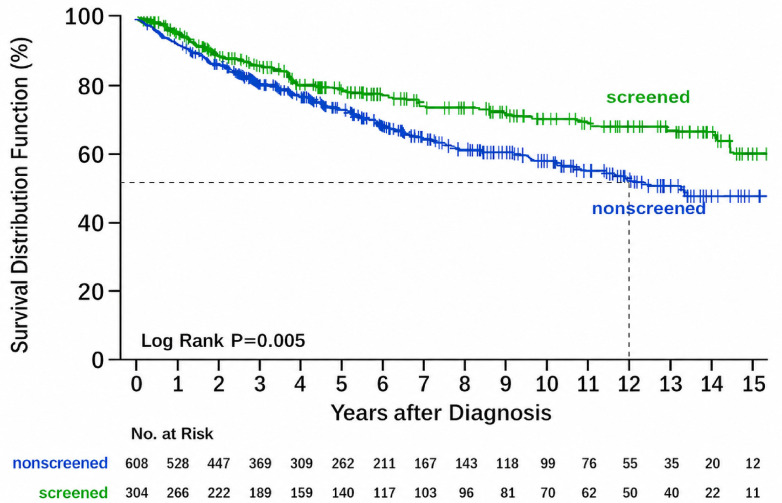
Kaplan–Meier overall survival curves for the matched screened and nonscreened groups.

Kaplan–Meier OS estimates at prespecified time points are presented in Table 3. Corresponding survival curves are presented in Figure 3. The screened group demonstrated consistently superior OS than did the nonscreened group throughout the follow-up period, with the survival gap progressively widening over time.

OS was 94.20% in the screened group and 93.41% in the nonscreened group at the 1-year follow-up and became increasingly divergent with longer follow-up. OS was 77.35% versus 71.80% at 5 years, 70.57% versus 58.99% at 9 years, and 58.38% versus 45.80% at 15 years. Median OS was not reached in the screened group. In the nonscreened group, median OS was 12.00 years (95% CI: 10.30—not estimable). The log-rank test confirmed a significant difference in unadjusted OS between the groups (*p* = 0.005). As shown in Figure 3, the progressively diverging survival curves suggest a clinically relevant long-term survival difference associated with prior FIT screening.

### 3.4. Results of Stage-Stratified OS Analysis

Stage-stratified Kaplan–Meier OS estimates are presented in Table 4. Among patients with early-stage disease (Stages 0–II), screened patients exhibited significantly better long-term OS than did nonscreened patients, with 5-year OS rates of 89.71% versus 82.09% and 15-year OS rates of 68.07% versus 52.24% (log-rank *p* = 0.001), indicating that the survival benefit of FIT screening was most pronounced among patients with earlier-stage disease.

Among patients with late-stage disease (Stages III and IV), screened patients had better OS at all time points. The screened and nonscreened groups had 5-year OS rates of 59.11% and 54.77% and 15-year OS of 42.45% and 35.07%, respectively; however, the between-group difference was nonsignificant (log-rank *p* = 0.317). Median OS among patients with late-stage disease was 9.01 years (95% CI: 4.93–NE) for screened patients and 5.92 years (95% CI: 4.52–NE) for nonscreened patients. Together, these stage-stratified findings suggest that the unadjusted OS advantage associated with FIT screening was more apparent among patients with early-stage disease than among those with late-stage disease, although residual confounding, lead-time bias, and differences in postdiagnosis care cannot be excluded.

### 3.5. Results of Age-Stratified OS Analysis

**Figure 4:**
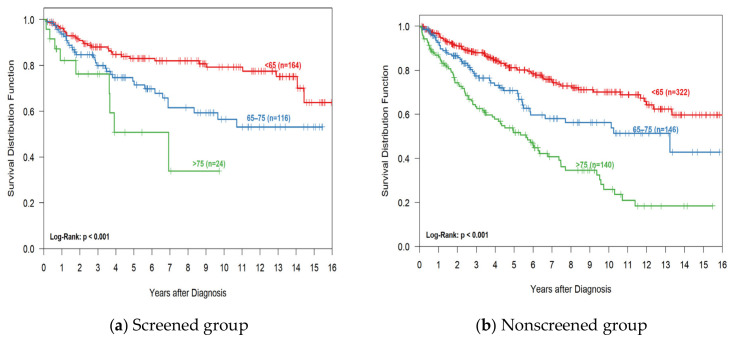
Age-stratified Kaplan–Meier overall survival curves for the matched screened and nonscreened groups. (**a**) Screened group. (**b**) Nonscreened group.

Age-stratified Kaplan–Meier OS estimates are presented in Table 5. Corresponding survival curves are presented in Figure 4. In both groups, OS declined with increasing age (both *p* = 0.001), consistent with expected age-related differences in competing mortality risks.

In the nonscreened group, 5-year OS rates were 81.25% for patients aged <65 years, 70.88% for those aged 65–75 years, and 51.74% for those aged >75 years; at 15 years, OS rates were 59.65%, 42.80%, and 18.39% in these age groups, respectively. In the screened group, a comparable age-dependent gradient was observed, with 5-year OS rates of 83.08%, 73.17%, and 50.86% across the same strata. Notably, screened patients aged 65–75 years (the core target of Taiwan’s national FIT program) had higher 15-year OS rates (53.12%) than their nonscreened counterparts (42.80%). Long-term estimates for screened patients aged >75 years (*n* = 24) warrant cautious interpretation given the small sample size and limited numbers at risk during later follow-up.

### 3.6. Prognostic Factors

**Figure 5:**
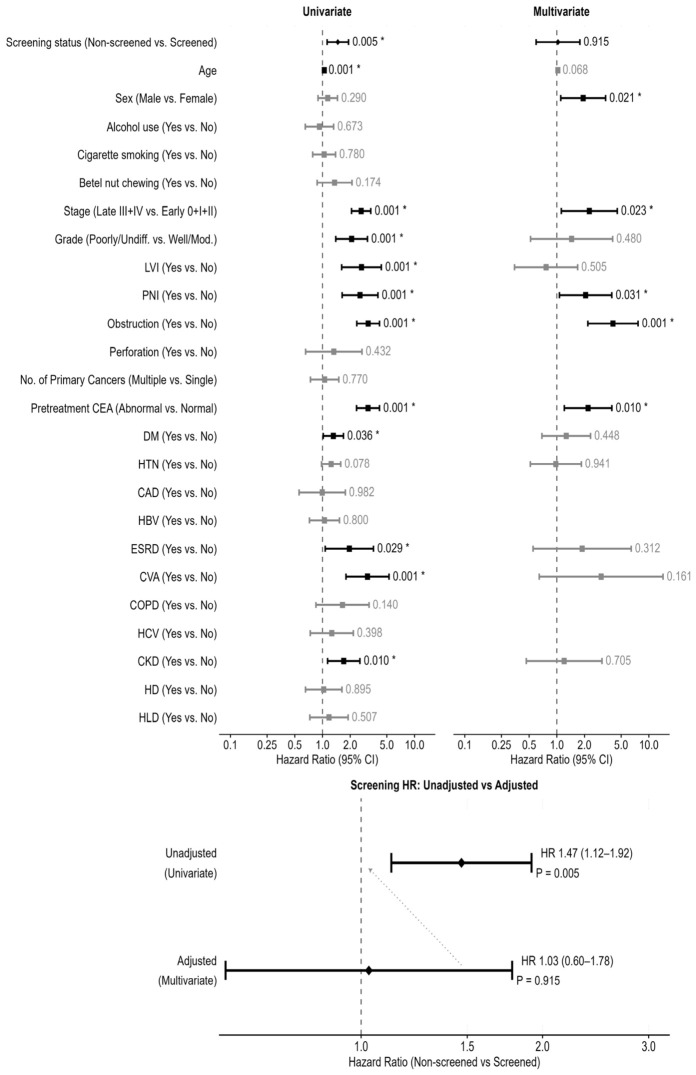
Results of univariate and multivariable regression analyses of overall survival in the propensity score-matched cohort. * *p* < 0.05.

Results of univariate and multivariable Cox proportional hazards regression analyses are presented in Table 6, and a forest plot of the results is presented in Figure 5.

In univariate analysis, nonscreened status was significantly associated with poorer OS (HR = 1.47; 95% CI: 1.12–1.92; *p* = 0.005). Additional adverse prognostic factors were older age (HR = 1.05; 95% CI: 1.03–1.06; *p* = 0.001), late-stage disease (HR = 2.63; 95% CI: 2.07–3.35; *p* = 0.001), poor or undifferentiated histological grade (HR = 2.07; 95% CI: 1.39–3.08; *p* = 0.001), positive LVI (HR = 2.65; 95% CI: 1.62–4.36; *p* = 0.001), positive PNI (HR = 2.56; 95% CI: 1.63–4.00; *p* = 0.001), tumor obstruction (HR = 3.13; 95% CI: 2.36–4.16; *p* = 0.001), abnormal pretreatment CEA level (HR = 3.12; 95% CI: 2.34–4.16; *p* = 0.001), diabetes mellitus (HR = 1.31; 95% CI: 1.02–1.69; *p* = 0.036), end-stage renal disease (HR = 1.96; 95% CI: 1.07–3.58; *p* = 0.029), cerebrovascular accident (HR = 3.08; 95% CI: 1.80–5.28; *p* = 0.001), and chronic kidney disease (HR = 1.70; 95% CI: 1.14–2.55; *p* = 0.010).

Variables with univariate *p* < 0.10 or established clinical relevance were entered into the multivariable model. Coronary artery disease was not included in the multivariable model because it showed no significant association with overall survival in univariate analysis. In the full multivariable model, FIT screening status was not independently associated with OS (HR = 1.03; 95% CI: 0.60–1.78; *p* = 0.915). Five independent adverse prognostic factors were identified: male sex (HR = 1.93; 95% CI: 1.10–3.39; *p* = 0.021), late-stage disease (HR = 2.25; 95% CI: 1.12–4.54; *p* = 0.023), positive PNI (HR = 2.06; 95% CI: 1.07–3.96; *p* = 0.031), tumor obstruction (HR = 4.07; 95% CI: 2.17–7.63; *p* = 0.001), and abnormal pretreatment CEA level (HR = 2.19; 95% CI: 1.21–3.96; *p* = 0.010). Notably, male sex, which was not significant in univariate analysis (HR = 1.14; 95% CI: 0.89–1.45; *p* = 0.290), emerged as a significant independent adverse prognostic factor after multivariable adjustment—a pattern consistent with a suppressor effect in which confounding by stage, obstruction, and pretreatment CEA level masked the sex–survival relationship in unadjusted analysis.

### 3.7. Reduced Multivariable Model and Sensitivity Analysis Results

**Figure 6:**
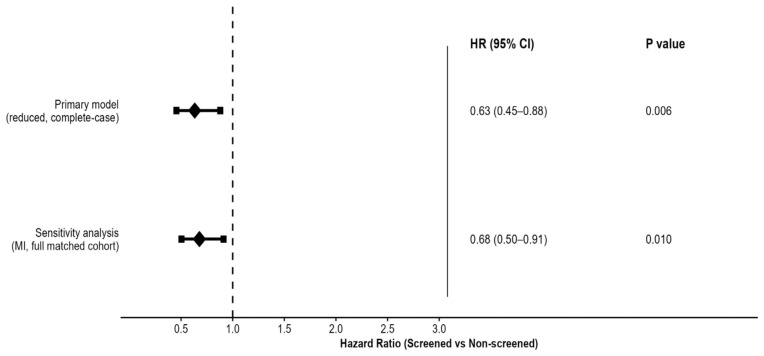
Results of multivariable Cox regression analysis of the effect of FIT screening status on overall survival in the propensity score-matched cohort.

To evaluate the robustness of the screening–survival association given the high rates of missing data for LVI (44–50% nonassessable) and PNI (54–55% nonassessable), a reduced multivariable Cox model was constructed, and a multiple imputation sensitivity analysis was conducted. Results are presented in Table 7, and a corresponding forest plot is presented in Figure 6.

In the primary reduced model—restricted to covariates with less than 20% missing data and excluding LVI and PNI (*n* = 731 for complete case analysis), FIT screening status was independently associated with significantly better OS after adjustment for age, sex, lifestyle factors, tumor stage, histological grade, obstruction, perforation, number of primary cancers, pretreatment CEA level, and comorbidities (HR = 0.63; 95% CI: 0.45–0.88; *p* = 0.006). In the multiple imputation sensitivity analysis restoring the full matched cohort of 912 patients, the association remained consistent and significant (HR = 0.68; 95% CI: 0.50–0.91; *p* = 0.010). This analysis reduced potential selection bias caused by complete-case exclusion, particularly for patients with nonassessable LVI, PNI, pretreatment CEA, or histological grade. The consistency between the complete-case reduced model and the multiple imputation model suggests that the observed association between FIT screening status and improved OS was not driven solely by missing pathological data.

The divergence between the full model (Table 6; HR = 1.03; *p* = 0.915) and the primary reduced model (Table 7; HR = 0.63; *p* = 0.006) warrants careful interpretation. The weakening of the FIT screening effect in the full model is likely because of the inclusion of downstream pathological features, such as LVI and PNI. LVI and PNI may represent downstream pathological features rather than pure baseline confounders, because screening-detected tumors are often identified at an earlier, more favorable disease stage before developing more aggressive invasive characteristics. A model that accounts for downstream pathological features can inadvertently obscure the clinical benefits of screening rather than remove genuine confounding. The consistency between the primary reduced model and the multiple imputation sensitivity analysis provides supportive evidence that FIT screening status was associated with better long-term OS in this regional hospital cohort, although the model-dependent nature of this association warrants cautious interpretation.

## 4. Discussion

This study examined the associations of FIT screening status with clinicopathological characteristics and long-term OS in patients with colorectal adenocarcinoma treated at a regional hospital in Taiwan. From a 1:2 propensity score-matched cohort of 912 patients, three key findings emerged. First, after stage-inclusive matching, the distribution of early-stage versus late-stage disease was comparable between the screened and nonscreened groups (62.90% vs. 61.30%; OR = 1.07; 95% CI: 0.81–1.43; *p* = 0.629), reflecting successful covariate balance by design rather than a test of population-level stage shift. Second, screened patients had consistently better long-term OS across all follow-up time points than did nonscreened patients, with 15-year OS rates of 58.38% versus 45.80% (log-rank *p* = 0.005); this advantage was significant among patients with early-stage disease (*p* = 0.001) but not among those with late-stage disease (*p* = 0.317). Third, the association between FIT screening and OS was model-dependent: FIT screening was not independently associated with OS in the full multivariable model incorporating LVI and PNI (HR = 1.03; *p* = 0.915), but was an independent favorable prognostic factor in the primary reduced model (HR = 0.63; 95% CI: 0.45–0.88; *p* = 0.006); this finding was further supported by the multiple imputation sensitivity analysis (HR = 0.68; 95% CI: 0.50–0.91; *p* = 0.010).

A key methodological consideration in the present study is the inclusion of tumor stage in the propensity score model. Because FIT screening may improve survival partly by detecting colorectal cancer at an earlier stage, tumor stage may lie on the causal pathway between screening and survival. Matching on stage can consequently attenuate or remove a substantial component of the total screening effect. For this reason, the present analysis should not be interpreted as estimating the overall population-level survival benefit of FIT screening. Rather, the stage-inclusive PSM design evaluates whether FIT screening status is independently associated with OS among patients with comparable stage distributions—a distinction critical for interpreting the observed survival difference. We adopted this stage-balanced comparison, rather than an estimate of the total, unadjusted screening effect, as the primary research question of this study, because the population-level stage-shift benefit of FIT screening in Taiwan has already been well documented in large national registry analyses [8,9,10], whereas institution-level evidence on whether FIT screening remains informative for survival after accounting for stage has been largely lacking. Because the final analytic dataset used in this study consisted of the propensity score-matched cohort, a reliable unmatched, stage-unadjusted comparison could not be reconstructed from the available data, and we were therefore unable to additionally report a crude survival analysis alongside the stage-balanced results; this constraint is acknowledged in the Limitations section, and the clinical significance of this stage-balanced association should accordingly be regarded as hypothesis-generating rather than confirmatory of an overall screening benefit.

Several methodological issues should be considered when interpreting the present findings. Stage was included in the propensity score model because it is a major determinant of colorectal cancer survival; however, stage may also represent a mediator between FIT screening and survival. Therefore, the stage-inclusive PSM design should be interpreted as a conservative stage-balanced comparison rather than as an estimate of the total causal effect of FIT screening. In addition, although PSM improved the comparability of several measured variables, residual imbalance remained for age group, sex, betel nut chewing, histological grade, pretreatment CEA level, and selected comorbidities. Accordingly, the observed survival difference should be interpreted cautiously as an adjusted observational association. To improve analytical transparency, standardized mean differences were reported, multivariable Cox regression was performed using both reduced and full models, multiple imputation sensitivity analysis was conducted for missing pathological data, and the proportional hazards assumption was assessed. These analyses support a cautious interpretation of the findings while avoiding overstatement of causal inference from a retrospective single-institution cohort.

Each of the aforementioned findings resonates with existing evidence. The comparable postmatching stage distribution is a methodologically expected outcome, given that stage was incorporated as a PSM covariate; this postmatching comparison therefore reflects a descriptive balance check rather than a population-level stage-shift test [19]. Consequently, the observed survival advantage associated with FIT screening cannot be attributed solely to earlier-stage detection.

Regarding survival outcomes, the progressively widening Kaplan–Meier curves across 15 years of follow-up are broadly consistent with national Taiwanese registry data indicating that FIT-based screening reduces CRC-specific mortality by up to 47% [9,10]. The results of stage-stratified analysis further suggest that the unadjusted survival advantage was more apparent among patients with early-stage disease than among those with late-stage disease; however, these results may be influenced by residual confounding, lead-time bias, and differences in postdiagnosis care [6,7]. A plausible clinical mechanism is that FIT screening detects occult lower gastrointestinal bleeding before symptom onset, prompting diagnostic colonoscopy at an earlier point in the disease course. Earlier endoscopic evaluation may identify tumors while they remain localized, resectable, and less likely to present with obstruction, extensive perineural or lymphovascular invasion, or other adverse pathological features. This mechanism may partly explain why the survival advantage of FIT screening was more pronounced in stage 0–II disease than in stage III–IV disease. The higher proportion of well-differentiated tumors in the screened group further suggests that FIT-detected cancers may harbor more favorable biological behavior, although this observation may also partly reflect length-time bias. Therefore, the stronger survival benefit observed in early-stage disease should be regarded as clinically meaningful but not exclusively causal, because screening-related biases and unmeasured differences in healthcare-seeking behavior may still have contributed.

Lead-time bias is particularly relevant because FIT screening may advance the date of CRC diagnosis, thereby lengthening the measured survival interval from diagnosis without necessarily delaying death. Length-time bias may have further contributed to the observed survival difference because FIT screening preferentially detects slower-growing, biologically less aggressive tumors with a longer preclinical detectable phase. The higher proportion of well-differentiated tumors in the screened group is consistent with this possibility and suggests that part of the OS advantage may reflect more favorable tumor biology rather than screening benefit per se. Although stage-inclusive PSM and stage-stratified analyses were used to mitigate crude stage imbalance, neither approach can fully eliminate lead-time or length-time bias. Therefore, the observed survival difference should be interpreted as an association between FIT screening status and improved OS rather than as definitive causal evidence that FIT screening alone prolonged survival.

To further characterize the potential contribution of lead-time bias, we used a Duffy-type correction logic as a bounded sensitivity framework, rather than as a fully parameterized patient-specific correction. This approach, recently formalized by Vratanar and Pohar Perme [20], is based on the principle that survival estimates after screen detection can be adjusted by subtracting an assumed sojourn time, namely the duration of the preclinical screen-detectable phase, from the observed survival time. Because our retrospective institutional registry did not capture individual-level sojourn-time data, interval-cancer status, or patient-specific screening-interval history, a formal patient-specific correction could not be performed. Instead, we conducted a literature-based bounded sensitivity assessment using a recent systematic review of the preclinical detectable phase for fecal-occult-blood-based colorectal cancer screening, which reported estimates of approximately 2 to 5 years [21]. Even under the conservative upper-bound assumption of a 5-year lead time, this interval accounts for only part of the 15-year follow-up period over which the Kaplan–Meier curves progressively diverged. Therefore, lead-time bias may have contributed to the observed OS difference, particularly during earlier follow-up, but is unlikely to fully explain the long-term separation of the survival curves.

Because the observed survival advantage persisted and continued to widen well beyond this plausible lead-time window, lead-time bias alone is unlikely to fully account for the magnitude of the observed OS difference, although it likely contributed to it, particularly during the earlier years of follow-up, and cannot be entirely excluded.

With respect to length-time bias, we agree that this bias cannot be fully eliminated without interval-cancer data. In the present study, we used stage-stratified survival analysis and between-group comparison of histological grade as practical approaches to partially characterize, rather than fully correct for, the potential influence of length-time bias. Because FIT screening may preferentially detect slower-growing tumors with a longer preclinical detectable phase, the higher proportion of well-differentiated tumors in the screened group is consistent with a possible length-time bias mechanism. Therefore, this histological-grade difference was interpreted cautiously and was discussed as a potential contributor to the observed survival association, rather than as definitive evidence of a true screening-related survival benefit. A fully parametric length-bias correction would require interval-cancer data, namely cancers diagnosed clinically between scheduled screening rounds, which were not available in our institutional registry and therefore could not be implemented.

Notably, two clinicopathological features differed significantly between the groups. Because both groups were balanced on stage through propensity score matching, this histological-grade difference should be interpreted as a stage-independent biological difference between screening-detected and non-screening-detected tumors, rather than a byproduct of residual stage imbalance. This observation is consistent with the hypothesis that FIT-based screening preferentially detects a biologically more indolent tumor subset—independent of, and in addition to, the well-established stage-shift mechanism. However, FIT screening status was not associated with significant differences in LVI, PNI, tumor obstruction, tumor perforation, pretreatment CEA level, molecular alteration status, or overall stage distribution after matching. The similar proportions of stage IV disease between groups suggest that the available stage-based indicator of metastatic burden was balanced in the matched cohort. Because tumor size and detailed metastatic burden data were not consistently recorded in the institutional registry, this study could not determine whether FIT screening was associated with smaller primary tumor size or lower metastatic volume. Therefore, the observed survival advantage should not be attributed solely to measured differences in tumor burden or invasive pathological features. Age-group distribution was another significant difference (*p* = 0.001), with a greater proportion of patients aged 65–75 years in the screened group (38.20% vs. 24.00%) and a greater proportion of patients aged >75 years in the nonscreened group (23.00% vs. 7.90%), consistent with the age eligibility criteria of Taiwan’s national FIT screening program.

Regarding the Cox regression findings, the attenuation of the FIT screening effect after full multivariable adjustment, particularly when LVI and PNI were included, may reflect adjustment for downstream pathological features rather than only baseline confounding; therefore, this model-dependent finding should be interpreted cautiously [22,23,24]. This asymmetry in handling stage and LVI/PNI—retaining stage as a matching covariate while treating LVI and PNI as downstream, missingness-driven exclusions from the primary model—reflects a design choice rather than an inconsistency in principle: both variables may act partly as mediators of the screening–survival relationship, and this shared limitation is discussed further below.

The five independent adverse prognostic factors identified in the full model (male sex, late-stage disease, positive PNI, tumor obstruction, and abnormal pretreatment CEA level) are each consistent with established evidence [25,26]. In particular, PNI is a widely recognized marker of aggressive tumor–nerve interaction and invasive biological behavior in colorectal cancer; previous studies have consistently demonstrated that PNI is associated with poorer survival, particularly in patients with stage II or locally advanced disease [22,23,24]. Tumor obstruction is an established adverse clinical feature because it often reflects advanced local tumor growth, emergency presentation, delayed diagnosis, and a higher likelihood of postoperative morbidity—all of which may contribute to inferior long-term outcomes [25]. Similarly, abnormal pretreatment CEA has long been regarded as an important prognostic biomarker. Elevated CEA may reflect greater tumor burden, occult metastatic potential, or biologically aggressive disease, and recent cohort evidence further supports its value in staging, risk stratification, and survival prediction [26]. In the present study, abnormal pretreatment CEA remained independently associated with poorer OS after multivariable adjustment, indicating that CEA provides prognostic information beyond screening status and tumor stage. From a clinical perspective, pretreatment CEA may help refine screening-based risk stratification: patients without prior FIT screening who also present with abnormal pretreatment CEA may represent a particularly high-risk subgroup requiring careful diagnostic staging, prompt treatment planning, and closer postdiagnosis surveillance. Conversely, FIT-screened patients with normal pretreatment CEA may reflect a more favorable risk profile, although this interpretation should be considered exploratory given the retrospective design. The adverse prognostic effects of PNI, tumor obstruction, and abnormal pretreatment CEA observed here are therefore clinically plausible and consistent with established colorectal cancer prognostic literature.

The emergence of male sex as an independent adverse prognostic factor only in the multivariable model is consistent with a suppressor effect, wherein confounding by stage and clinical variables masked the sex–survival relationship in unadjusted analysis [25]. Once these adverse clinical features were adjusted for, the underlying sex-based survival disadvantage became apparent, consistent with the known prognostic significance of sex in multivariable CRC survival analyses [25]. Molecular comparisons of *BRAF*, *KRAS*, and *NRAS* mutation status should be interpreted cautiously given stage-dependent ascertainment bias from selective testing, predominantly in patients with advanced disease; comprehensive *BRAF*, *KRAS*, and *NRAS* testing in patients with metastatic CRC remains a key part of European Society For Medical Oncology guidelines [27].

The most important contribution of this study is that it provides real-world institutional evidence from a regional hospital setting, complementing large-scale national registry findings from Taiwan [8,9,10]. By applying 1:2 PSM alongside both a prespecified primary reduced model and a multiple imputation sensitivity analysis, this study explicitly addressed two methodological challenges frequently underreported in single-institution CRC cohort studies: confounding by measured baseline differences and attrition bias from missing pathological data. The primary reduced model retained 731 of 912 matched patients (80.2%), and the multiple imputation analysis incorporated all 912 patients, substantially mitigating selection bias concerns. The consistency of findings across both analytical approaches provides additional supportive evidence beyond a single complete-case analysis alone. Furthermore, by showing that the screening–survival association differed according to whether LVI and PNI were included in the multivariable model, this study highlights the importance of carefully distinguishing baseline confounders from downstream pathological features in regional hospital-based CRC outcome studies [8,9,10,28]. The findings underscore the need to optimize FIT participation, ensure timely colonoscopy follow-up, and perform thorough pathological staging across diverse rural–urban populations. Beyond methodological transparency, identifying a survival association that persists after stage balancing raises the possibility that these differences are screening-related. However, none of these factors was directly measured in the present dataset, and the residual association could equally reflect unmeasured confounding or a healthy screenee effect rather than a genuine biological or behavioral difference attributable to screening itself; the present observational design does not allow these explanations to be distinguished. Should future studies with more granular, directly measured data confirm that such differences genuinely track with screening status, these factors could become clinically actionable through targeted follow-up and supportive care independent of stage shift; at present, however, this possibility should be regarded as hypothesis-generating rather than an established basis for clinical practice.

Building on these clinically actionable factors, emerging digital approaches may further support postdiagnosis risk stratification and follow-up care in FIT-screened populations. Recent advances in computational pathology and e-health interventions further highlight the potential role of digital tools in cancer risk stratification, biomarker-based outcome prediction, and patient-centered survivorship care [28,29]. Although these approaches were beyond the scope of the present study, they may complement FIT-based screening and postdiagnosis risk assessment in future colorectal cancer care models.

Several limitations warrant acknowledgment. First, the retrospective single-institution design limits causal inference and generalizability. Because this study was conducted in a Taiwanese regional hospital within an organized national FIT-based screening program, the findings may not be directly generalizable to healthcare systems with different screening infrastructures, eligibility criteria, colonoscopy accessibility, referral pathways, or follow-up mechanisms. Population characteristics—including age distribution, rural–urban composition, health literacy, socioeconomic background, and healthcare-seeking behavior—may also differ across countries and regions. Therefore, the observed association between FIT screening and improved OS should be interpreted within the context of Taiwan’s regional healthcare setting, and external validation in multicenter or population-based cohorts from other healthcare systems is warranted.

Second, residual confounding from unmeasured variables may remain. Although several comorbidities—including diabetes mellitus, hypertension, coronary artery disease, hepatitis B and C, end-stage renal disease, cerebrovascular accident, chronic obstructive pulmonary disease, chronic kidney disease, heart disease, and hyperlipidemia—were available and incorporated into the analysis, information on socioeconomic status, healthcare access, health literacy, colonoscopy follow-up interval, dietary habits, family history, functional status, obesity, and other lifestyle or metabolic factors was not consistently recorded in the institutional registry. Therefore, screened patients may have differed from nonscreened patients in unmeasured nonclinical characteristics, including greater health awareness, better access to preventive services, and higher adherence to follow-up care. These differences may have contributed to the observed survival advantage and should be considered when interpreting the association between FIT screening and overall survival.

Third, although PSM was performed using age, sex, and stage as covariates, residual imbalance persisted after matching. Age-group distribution remained significantly imbalanced (SMD = 0.305), with a higher proportion of patients aged >75 years in the nonscreened group. Re-matching using a stricter caliper width or exact age-category matching was considered as a means of reducing this imbalance; however, because the >75-year stratum already contained a limited number of screened patients (*n* = 24), further restricting the matching criteria would have disproportionately reduced sample size and statistical precision in this subgroup. We therefore retained the original matching scheme and instead relied on adjustment for continuous age in the multivariable Cox model and prespecified age-stratified sensitivity analyses to mitigate residual age-related confounding. This residual imbalance should be regarded as a limitation of the present analysis despite the mitigation strategies described above. Nonetheless, because the age-stratified survival estimates were directionally consistent across all three age groups (Table 5, Figure 4), we consider this consistency to constitute an empirical robustness check of our conclusions with respect to the residual age imbalance, in lieu of re-performing propensity score matching. As an additional sensitivity approach, Inverse Probability of Treatment Weighting (IPTW) applied to the full unmatched cohort could be used in future analyses to further verify the robustness of the observed FIT-screening association without discarding unmatched patients; we identify this as a concrete direction for subsequent work, particularly given the small size of the >75-year screened subgroup that limited weight stability in the present dataset. Sex distribution was also marginally imbalanced after matching, and male sex was identified as an independent adverse prognostic factor in multivariable analysis. In addition, because the final analytic dataset consisted of the propensity score-matched cohort, complete baseline characteristics prior to matching could not be reliably reconstructed; accordingly, group balance was assessed primarily based on postmatching characteristics. Additionally, betel nut chewing demonstrated a residual SMD of 0.123 after propensity score matching, exceeding the conventional balance threshold of 0.10. Although betel nut chewing was not significantly associated with overall survival in univariate analysis and was retained as a covariate in the multivariable models, its potential residual confounding effect on survival outcomes cannot be entirely excluded.

Fourth, several lifestyle-related and clinical variables were incompletely captured. Obesity is an established risk factor for colorectal cancer and may also influence survival outcomes; however, body mass index, body weight, body height, and obesity status were not consistently available in the institutional registry and therefore could not be included as a baseline characteristic or covariate in the PSM or multivariable survival models. Similarly, alcohol use and cigarette smoking were recorded only as categorical variables (yes/no). Detailed quantitative exposure data—including ethanol amount, duration of alcohol consumption, daily cigarette consumption, and smoking duration—were not consistently recorded. As a result, objective indices such as cumulative ethanol exposure and the Brinkman index could not be calculated, and residual confounding related to lifestyle exposure intensity may remain.

Fifth, the observed survival advantage may reflect multiple mechanisms, including earlier clinical detection, more favorable tumor biology, better adherence to diagnostic follow-up and treatment, and differences in general health status between screened and nonscreened patients. However, residual confounding, competing mortality, and a healthy screenee effect cannot be excluded. Screened individuals may have been more health-conscious, more adherent to medical follow-up, and more proactive in seeking timely care, whereas the nonscreened group included a larger proportion of older patients at higher risk of non-CRC mortality. Reliable cause-of-death information was not consistently available in the institutional registry; therefore, CRC-specific death and CRC-specific survival could not be directly evaluated. Although FIT screening remained associated with improved OS in the reduced multivariable model and multiple imputation analysis, this association should be interpreted cautiously as an adjusted observational finding rather than definitive evidence of a causal survival benefit.

Sixth, because tumor stage was included in the propensity score model, the matched analysis may have adjusted for a potential mediator of the screening–survival relationship. Therefore, the observed association should be interpreted as the survival difference associated with FIT screening status among patients with similar stage distributions, not as the total causal survival benefit of FIT screening. This limitation is conceptually analogous to the downstream nature of LVI and PNI discussed above (Section 3.7 and Section 4): tumor stage, like LVI and PNI, may only partly represent a baseline confounder and may partly reflect a consequence of earlier detection through screening. Unlike LVI and PNI, however, stage could not be omitted from the propensity score model because it was essential for establishing baseline comparability between screened and nonscreened groups, and its near-complete ascertainment made it feasible for matching. Readers should therefore interpret both the stage-matched design and the LVI/PNI-excluded reduced model as complementary strategies for approximating, rather than fully isolating, the baseline confounding structure in this observational cohort.

FIT exposure may also have been misclassified because tests performed outside the hospital system may not have been completely captured. Furthermore, no fixed time window between the FIT date and the cancer diagnosis date was defined to qualify screening exposure; therefore, the possibility that a minority of FIT tests were prompted by early subclinical symptoms—rather than representing true asymptomatic population-based screening—cannot be entirely excluded. This study also could not distinguish single from repeated FIT participation; consequently, cumulative screening exposure, which has been reported to exhibit a dose–response relationship with survival benefit [9], could not be evaluated.

Seventh, several pathological, molecular, treatment-related, and metastatic burden variables were incompletely available. LVI and PNI were nonassessable in approximately 44–55% of patients, and molecular testing was performed selectively, introducing stage-dependent ascertainment bias. Treatment details—including surgical approach, adjuvant chemotherapy regimen, and surveillance intensity—were not captured. Tumor size and detailed metastatic burden indicators, including the number, size, and anatomical distribution of metastatic lesions, were not consistently available in the institutional registry. Although stage (particularly stage IV disease) served as the available proxy for metastatic disease burden, it could not fully capture metastatic volume or metastatic site-specific differences. A formal stage-stratified comparison of histological grade and other pathological features (i.e., comparing screened and nonscreened patients separately within each stage stratum) was not performed in the present analysis. Such stratified analysis could further confirm whether the observed stage-independent biological difference is consistent across individual stage strata and represents a valuable direction for future study.

Finally, lead-time bias and length-time bias cannot be completely excluded [30]. Because survival time was measured from the date of CRC diagnosis, earlier detection through FIT may have prolonged the observed OS interval without necessarily altering the natural course of disease. Moreover, FIT screening may preferentially identify slower-growing tumors with more favorable biological behavior, which could partly account for the better long-term OS observed in the screened group. As detailed in the Discussion section, a bounded sensitivity analysis using a recently published, colorectal-cancer-specific range of preclinical detectable phase duration indicated that the magnitude and persistence of the survival advantage across the 15-year follow-up period exceed what lead-time bias alone would be expected to produce, although a partial contribution cannot be excluded [20,21].

Future prospective studies are needed to more precisely characterize how organized FIT screening translates into long-term survival benefit in regional hospital-based CRC cohorts. Such studies should incorporate cumulative FIT participation records, validated cause-of-death data, complete pathological staging, treatment details, and bias-corrected survival methods. Beyond these foundational elements, integrating artificial intelligence-based pathological biomarkers—such as deep learning models for predicting microsatellite instability or tumor mutational burden [29]—may further refine FIT-based risk stratification, while structured digital follow-up interventions could help improve colonoscopy adherence and patient-centered outcomes among FIT-positive individuals [31].

## 5. Conclusions

In this propensity score-matched cohort of patients with colorectal adenocarcinoma treated at a regional hospital in Taiwan, FIT-screened patients demonstrated significantly better 15-year OS than nonscreened patients (58.38% vs. 45.80%; log-rank *p* = 0.005). FIT screening status was independently associated with improved OS in both the primary reduced model (HR = 0.63; *p* = 0.006) and the multiple imputation sensitivity analysis (HR = 0.68; *p* = 0.010). Because stage was included in the propensity score model, these findings should be interpreted as an association within a stage-balanced cohort rather than as evidence of the total causal survival benefit of FIT screening. Therefore, although FIT screening was associated with improved overall survival after propensity score matching, this finding should be interpreted cautiously because CRC-specific survival was unavailable and residual age-related imbalance remained after matching, particularly among patients aged >75 years (23.00% vs. 7.90%), which may have contributed to the observed survival difference. The survival advantage was most pronounced among patients with early-stage disease. These findings highlight the real-world value of organized FIT screening programs in regional hospital settings, where optimizing participation rates, ensuring timely colonoscopy follow-up after a positive test, and achieving complete pathological staging remain critical to improving CRC outcomes. Future studies incorporating validated cause-of-death data are needed to determine whether FIT screening is independently associated with improved CRC-specific survival.

## Figures and Tables

**Table 1:** Postmatching baseline clinicopathological characteristics in the propensity score-matched cohort.

Variables		Screened (*n* = 304)		Nonscreened (*n* = 608)		*p* Value	SMD
		*n*	%	*n*	%		
Age, mean ± SD (min–max)		63.40 ± 8.80 (42–91)		63.50 ± 13.5 (20–97)		0.880	0.009
Age group						0.001 *	0.305
	<65	164	53.90	322	53.00		
	65–75	116	38.20	146	24.00		
	>75	24	7.90	140	23.00		
Sex						0.061	0.138
	Female	113	37.20	267	43.90		
	Male	191	62.80	341	56.10		
Alcohol use						0.866	0.019
	No	260	85.50	524	86.20		
	Yes	44	14.50	84	13.80		
Betel nut chewing						0.096	0.123
	No	278	91.40	575	94.60		
	Yes	26	8.60	33	5.40		
Cigarette smoking						0.228	0.090
	No	228	75.00	479	78.80		
	Yes	76	25.00	129	21.20		
Primary Site						0.811	0.022
	Proximal	78	25.70	162	26.60		
	Distal	226	74.30	446	73.40		
Grade						0.001 *	0.158
	Well differentiated	92	30.30	119	19.60		
	Moderately differentiated	167	54.90	371	61.00		
	Poorly differentiated	18	5.90	26	4.30		
	Undifferentiated	2	0.70	3	0.50		
	Unknown	25	8.20	89	14.60		
Pretreatment CEA						0.077	0.114
	Normal	142	46.70	255	41.90		
	Abnormal	81	26.60	207	34.00		
	Not assessable/Not available	81	26.60	146	24.00		
BRAF						0.553	0.050
	Wild-type	113	37.20	226	37.20		
	Mutated	19	6.20	50	8.20		
	Not assessable/Not available	172	56.60	332	54.60		
KRAS						0.614	0.056
	Wild-type	92	30.30	202	33.20		
	Mutated	7	2.30	16	2.60		
	Not assessable/Not available	205	67.40	390	64.10		
NRAS						0.400	0.077
	Wild-type	48	15.80	116	19.10		
	Mutated	2	0.70	6	1.00		
	Not assessable/Not available	254	83.60	486	79.90		
Obstruction						0.759	0.033
	No	245	80.60	486	79.90		
	Yes	36	11.80	81	13.30		
	Not assessable/Not available	23	7.60	41	6.70		
Perforation						0.148	0.088
	No	277	91.10	549	90.30		
	Yes	3	1.00	18	3.00		
	Not assessable/Not available	24	7.90	41	6.70		
Perineural invasion						0.447	0.057
	No	89	29.30	188	30.90		
	Yes	51	16.80	83	13.70		
	Not assessable/Not available	164	53.90	337	55.40		
Lymphovascular invasion						0.145	0.098
	No	85	28.00	136	22.40		
	Yes	84	27.60	169	27.80		
	Not assessable/Not available	135	44.40	303	49.80		
Stage						0.413	0.073
	0	22	7.20	34	5.60		
	I	105	34.50	181	29.80		
	II	63	20.70	155	25.50		
	III	73	24.00	161	26.50		
	IV	39	12.80	73	12.00		
	Unknown	2	0.70	4	0.70		
DM						0.136	0.110
	No	210	69.10	450	74.00		
	Yes	94	30.90	158	26.00		
HTN						0.152	0.106
	No	132	43.40	296	48.70		
	Yes	172	56.60	312	51.30		
CAD						0.181	0.102
	No	284	93.40	582	95.70		
	Yes	20	6.60	26	4.30		
HBV						0.437	0.062
	No	266	87.50	519	85.40		
	Yes	38	12.50	89	14.60		
ESRD						0.287	0.088
	No	295	97.00	598	98.40		
	Yes	9	3.00	10	1.60		
CVA						0.332	0.088
	No	299	98.40	590	97.00		
	Yes	5	1.60	18	3.00		
COPD						0.682	0.047
	No	299	98.40	594	97.70		
	Yes	5	1.60	14	2.30		
HCV						0.867	0.023
	No	289	95.10	581	95.60		
	Yes	15	4.90	27	4.40		
CKD						0.136	0.111
	No	276	90.80	570	93.80		
	Yes	28	9.20	38	6.20		
HD						0.362	0.072
	No	276	90.80	564	92.80		
	Yes	28	9.20	44	7.20		
HLD						0.126	0.113
	No	274	90.10	567	93.30		
	Yes	30	9.90	41	6.70		
Number of Primary Cancers						0.396	0.086
	1	273	89.80	523	86.00		
	2	29	9.50	77	12.70		
	3	2	0.70	7	1.20		
	4	0	0.00	1	0.20		

SMD, standardized mean difference. SMD < 0.10 indicates adequate covariate balance after propensity score matching. Variables with SMD ≥ 0.10 indicated residual imbalance after matching. These included age group, sex, betel nut chewing, histological grade, pretreatment CEA level, and selected comorbidities. Clinically relevant variables and variables associated with overall survival were therefore considered in the multivariable Cox regression models to further reduce potential residual confounding. * *p* < 0.05.

**Table 2:** Distribution of early- and late-stage disease in the propensity score-matched cohort.

Stage Category ^1^	Screened *n* = 302	Nonscreened *n* = 604	Total *n* = 906	*p* Value	OR (95% CI)
Early Stage 0 + I + II	190 (62.90)	370 (61.30)	560 (61.80)	0.629	—
Late Stage III + IV	112 (37.10)	234 (38.70)	346 (38.20)		1.07 (0.81–1.43)

^1^ Patients with unknown stage or unavailable stage data were excluded; therefore, the denominator for this stage-category analysis differs from that of the full matched cohort. The odds ratio (OR) represents the risk of late-stage disease (Stages III and IV) in nonscreened patients compared with that in screened patients. OR > 1 indicates higher odds of late-stage disease in the nonscreened group than in the screened group. Because stage was included in the propensity score model, this postmatching stage comparison should be interpreted as a descriptive balance assessment rather than as a test of a population-level stage-shift effect. Abbreviations: OR, odds ratio; CI, confidence interval; PSM, propensity score matching. — indicates the reference category.

**Table 3:** Kaplan–Meier estimates of overall survival in the matched screened and nonscreened groups.

Group	Screened	Nonscreened	*p* Value
Time Point	(*n* = 304)	(*n* = 608)	
1-year	94.20	93.41	0.005 *
3-year	84.17	79.85	
5-year	77.35	71.80	
7-year	72.12	63.06	
9-year	70.57	58.99	
11-year	67.72	53.21	
13-year	64.98	48.80	
15-year	58.38	45.80	
Median OS, years (95% CI)	NE	12.00 (10.30–NE)	

* *p* value represents log-rank test for overall comparison.

**Table 4:** Stage-stratified Kaplan–Meier estimates of overall survival in the matched screened and nonscreened groups.

Stage	Early Stage 0 + I + II			Late Stage III + IV		
Group Time	Screened (*n* = 190)	Nonscreened (*n* = 370)	*p* Value	Screened (*n* = 112)	Nonscreened (*n* = 234)	*p* Value
1-year	95.60	96.12	0.001 *	91.74	89.46	0.317
3-year	92.15	88.87		69.73	65.23	
5-year	89.71	82.09		59.11	54.77	
7-year	84.95	73.21		53.34	45.88	
9-year	83.65	68.10		51.50	43.58	
11-year	82.23	59.89		46.69	42.08	
13-year	82.10	54.51		42.45	39.45	
15-year	68.07	52.24		42.45	35.07	
Median OS, years (95% CI)	NE	NE		9.01 (4.93–NE)	5.92 (4.52–NE)	

* *p* < 0.05.

**Table 5:** Age-stratified Kaplan–Meier estimates of overall survival in the matched screened and nonscreened groups.

Group	Screened				Nonscreened			
Age Time	<65 (*n* = 164)	65–75 (*n* = 116)	>75 (*n* = 24)	*p* Value	<65 (*n* = 322)	65–75 (*n* = 146)	>75 (*n* = 140)	*p* Value
1-year	96.24	93.72	82.17	0.001 *	96.82	92.76	87.00	0.001 *
3-year	88.10	80.01	76.30		88.06	77.55	63.43	
5-year	83.08	73.17	50.86		81.25	70.88	51.74	
7-year	82.03	61.61	33.91		75.21	58.07	40.70	
9-year	80.75	59.41	33.91		71.24	56.26	34.67	
11-year	79.30	56.44	—		68.98	51.36	21.01	
13-year	75.16	53.12	—		62.36	51.36	18.39	
15-year	63.77	53.12	—		59.65	42.80	18.39	

^—^ Insufficient number at risk for stable estimation. * *p* < 0.05.

**Table 6:** Prognostic factors for overall survival in the propensity score-matched cohort.

Variable	Univariate Analysis		Multivariable Analysis	
	HR (95% CI)	*p* Value	HR (95% CI)	*p* Value
Screening status (Nonscreened vs. Screened)	1.47 (1.12–1.92)	0.005 *	1.03 (0.60–1.78)	0.915
Age	1.05 (1.03–1.06)	0.001 *	1.03 (1.00–1.05)	0.068
Sex (Male vs. Female)	1.14 (0.89–1.45)	0.290	1.93 (1.10–3.39)	0.021 *
Alcohol use (Yes vs. No)	0.93 (0.65–1.32)	0.673	—	—
Cigarette smoking (Yes vs. No)	1.04 (0.78–1.39)	0.780	—	—
Betel nut chewing (Yes vs. No)	1.35 (0.87–2.09)	0.174	—	—
Stage (Late III + IV vs. Early 0 + I + II)	2.63 (2.07–3.35)	0.001 *	2.25 (1.12–4.54)	0.023 *
Grade (Poorly/Undiff. vs. Well/Mod.)	2.07 (1.39–3.08)	0.001 *	1.45 (0.52–4.04)	0.480
LVI (Yes vs. No)	2.65 (1.62–4.36)	0.001 *	0.76 (0.35–1.68)	0.505
PNI (Yes vs. No)	2.56 (1.63–4.00)	0.001 *	2.06 (1.07–3.96)	0.031 *
Obstruction (Yes vs. No)	3.13 (2.36–4.16)	0.001 *	4.07 (2.17–7.63)	0.001 *
Perforation (Yes vs. No)	1.33 (0.66–2.69)	0.432	—	—
No. of Primary Cancers (Multiple vs. Single)	1.05 (0.74–1.50)	0.770	—	—
Pretreatment CEA (Abnormal vs. Normal)	3.12 (2.34–4.16)	0.001 *	2.19 (1.21–3.96)	0.010 *
DM (Yes vs. No)	1.31 (1.02–1.69)	0.036 *	1.27 (0.69–2.32)	0.448
HTN (Yes vs. No)	1.24 (0.98–1.58)	0.078	0.98 (0.52–1.85)	0.941
CAD (Yes vs. No)	0.99 (0.56–1.77)	0.982	—	—
HBV (Yes vs. No)	1.05 (0.72–1.53)	0.800	—	—
ESRD (Yes vs. No)	1.96 (1.07–3.58)	0.029 *	1.88 (0.55–6.43)	0.312
CVA (Yes vs. No)	3.08 (1.80–5.28)	0.001 *	3.03 (0.64–14.26)	0.161
COPD (Yes vs. No)	1.65 (0.85–3.21)	0.140	—	—
HCV (Yes vs. No)	1.26 (0.74–2.16)	0.398	—	—
CKD (Yes vs. No)	1.70 (1.14–2.55)	0.010 *	1.20 (0.47–3.10)	0.705
HD (Yes vs. No)	1.03 (0.65–1.63)	0.895	—	—
HLD (Yes vs. No)	1.18 (0.73–1.90)	0.507	—	—

Note: “—” indicates that the variable was not entered into the multivariable model because it did not meet the prespecified inclusion criterion of *p* < 0.10 in univariate analysis or was not retained after consideration of model stability and clinical relevance. Coronary artery disease (CAD) was not included in the multivariable model because of its nonsignificant association with overall survival in univariate analysis. * *p* < 0.05.

**Table 7:** Results of multivariable Cox regression analysis of overall survival in the propensity score-matched cohort.

Analysis	Screened (*n*)	Nonscreened (*n*)	Cox Sample (*n*)	HR (95% CI)	*p* Value
Primary model (reduced, complete-case)	304	608	731	0.63 (0.45–0.88)	0.006 *
Sensitivity analysis (MI, full matched cohort)	304	608	912 (imputed)	0.68 (0.50–0.91)	0.010 *

Note: The proportional hazards assumption was assessed using Schoenfeld residuals. The global Schoenfeld residual test was not statistically significant for the reduced Cox model (global *p* = 0.281), although stage and tumor obstruction showed possible time-dependent effects. Because the global test was nonsignificant and no time-dependent effect was observed for the main exposure variable, FIT screening status (*p* = 0.138), the standard Cox proportional hazards model was considered acceptable for the primary analysis. Primary model: complete-case analysis restricted to covariates with <20% missing data (LVI and PNI) were excluded. CAD was additionally excluded because it showed no significant association with overall survival in univariate analysis (HR = 0.99; *p* = 0.982). Sensitivity analysis: the mice package in R (*m* = 20, predictive mean matching) was used to impute missing values for LVI, PNI, CEA, and grade; results were pooled using Rubin’s rules. The imputation model included all variables used in the Cox regression model, together with survival time and survival status, to preserve the relationship between covariates and the outcome. Both analyses included age, sex, alcohol use, betel nut chewing, cigarette smoking, tumor stage, histological grade, obstruction, perforation, number of primary cancers, pretreatment CEA level, and comorbidities. Ties were handled using the Breslow method. * *p* < 0.05.

## Data Availability

The datasets generated and/or analyzed during the present study are not publicly available but may be available from the corresponding author upon reasonable request.

## Abbreviations

The following abbreviations are used in this manuscript:

AJCC	American Joint Committee on Cancer
BRAF	B-Raf Proto-Oncogene, Serine/Threonine Kinase
CAD	Coronary Artery Disease
CEA	Carcinoembryonic Antigen
CI	Confidence Interval
CKD	Chronic Kidney Disease
COPD	Chronic Obstructive Pulmonary Disease
CRC	Colorectal Cancer
CVA	Cerebrovascular Accident
DM	Diabetes Mellitus
ESRD	End-Stage Renal Disease
FIT	Fecal Immunochemical Test
HBV	Hepatitis B Virus
HCV	Hepatitis C Virus
HD	Heart Disease
HLD	Hyperlipidemia
HR	Hazard Ratio
HTN	Hypertension
IRB	Institutional Review Board
KRAS	Kirsten Rat Sarcoma Virus Oncogene Homolog
LVI	Lymphovascular Invasion
MI	Multiple Imputation
NE	Not Estimable
NRAS	Neuroblastoma RAS Viral Proto-Oncogene Homolog
OR	Odds Ratio
OS	Overall Survival
PNI	Perineural Invasion
PSM	Propensity Score Matching
RAS	Rat Sarcoma Viral Proto-Oncogene
SD	Standard Deviation
SMD	Standardized Mean Differences
